# Tumor-Targeted Delivery of IL-2 by NKG2D Leads to Accumulation of Antigen-Specific CD8+ T Cells in the Tumor Loci and Enhanced Anti-Tumor Effects

**DOI:** 10.1371/journal.pone.0035141

**Published:** 2012-04-11

**Authors:** Tae Heung Kang, Chih-Ping Mao, Liangmei He, Ya-Chea Tsai, Katherine Liu, Victor La, T.-C. Wu, Chien-Fu Hung

**Affiliations:** 1 Department of Pathology, Johns Hopkins University, Baltimore, Maryland, United States of America; 2 Department of Oncology, Johns Hopkins University, Baltimore, Maryland, United States of America; 3 Department of Obstetrics and Gynecology, Johns Hopkins University, Baltimore, Maryland, United States of America; 4 Molecular Microbiology and Immunology, Johns Hopkins University, Baltimore, Maryland, United States of America; Carl-Gustav Carus Technical University-Dresden, Germany

## Abstract

Interleukin-2 (IL-2) has been shown to promote tumor-specific T-cell proliferation and differentiation but systemic administration of IL-2 results in significant toxicity. Therefore, a strategy that can specifically deliver IL-2 to the tumor location may alleviate concerns of toxicity. Because NKG2D ligands have been shown to be highly expressed in many cancer cells but not in healthy cells, we reason that a chimeric protein consisting of NKG2D linked to IL-2 will lead to the specific targeting of IL-2 to the tumor location. Therefore, we created chimeric proteins consisting of NKG2D linked to *Gaussia* luciferase (GLuc; a marker protein) or IL-2 to form NKG2D-Fc-GLuc and NKG2D-Fc-IL2, respectively. We demonstrated that NKG2D linked to GLuc was able to deliver GLuc to the tumor location *in vivo*. Furthermore, we showed that TC-1 tumor-bearing mice intramuscularly injected with DNA encoding NKG2D-Fc-IL2, followed by electroporation, exhibited an increased number of luciferase-expressing E7-specific CD8+ T cells at the tumor location. More importantly, treatment with the DNA construct encoding NKG2D-Fc-IL2 significantly enhanced the therapeutic anti-tumor effects generated by intradermal vaccination with therapeutic HPV DNA in tumor-bearing mice. Therefore, by linking NKG2D to IL2, we are able to specifically deliver IL-2 to the tumor location, enhancing antigen-specific T-cell immune response and controlling tumor growth. Our approach represents a platform technology to specifically deliver proteins of interest to tumor loci.

## Introduction

Conventional chemotherapy and radiotherapy treatments are commonly plagued with toxic side effects since they are often not developed with high specificity to cancer cells. That is, both healthy and cancerous cells are affected, resulting in significant side effects and toxicity. Therefore, there is an urgent need for tumor-specific treatments that will be more effective than the conventional therapies in specifically targeting the tumorigenic cells, while not jeopardizing healthy organs and/or the well-being of the patient [1]. In this regard, antigen-specific T cell immunotherapy for cancer has emerged as a promising approach for specifically targeting tumor cells but not normal cells.

One common approach for antigen-specific T cell immunotherapy is to use *ex vivo* expansion of antigen-specific T cells with their subsequent transfer to the patient. Several approaches have been used to improve the antigen specificity of T cells, such as *in vitro* stimulation of the T cell by antigen-pulsed dendritic cells. Alternatively T cells can be transduced with a chimeric antigen receptor that can activate T cells through the T cell signaling pathway while bestowing the T cell with tumor specificity (for reviews, see [2], [3], [4]). Many of these approaches using adoptive transfer of antigen-specific CD8+ T cells require the administration of IL-2.

Interleukin-2 (IL-2) is a cytokine from the cytokine-receptor γ-chain family with many functions including stimulating the proliferation of T cells, inducing the production of NK cells, inducing cytotoxic T lymphocyte generation, and facilitating the proliferation and synthesis of immunoglobulins produced by B cells [5]. IL-2 induces effects by binding to pre-formed high-affinity heterotrimeric IL-2 receptors at the surface of activated cells. Because of its functional versatility, IL-2 has previously been used in experiments to augment the immune system [6]. It has also been shown that activated T cells can be supported by transgenic expression of IL-2 *in vivo* [7], [8] and IL-2 has already been approved for clinical use in patients with metastatic renal-cell carcinoma and malignant melanoma [5]. However, when administered systemically in high doses, tumor regression comes with transient side effects ranging from general malaise, fever, and nausea and vomiting to more severe hepatic dysfunction, increased capillary permeability, and decreased systemic vascular resistance [9], [10]. Thus, a strategy that is able to specifically deliver IL-2 to the tumor location will significantly reduce the required amount of administered IL-2, alleviating many of the side effects commonly associated with its systemic administration.

Identifying surface molecules that are specifically expressed in tumor cells but not in normal cells will be useful for the specific delivery of IL-2 to the tumor loci. For example, multiple NKG2D ligands have been identified and are known to be upregulated in transformed, infected, and/or stressed cells but not in substantial amounts in healthy adult cells [11]. Cellular and genotoxic stresses, like excessive proliferation, heat shock, or oxidative stress, are just some of the factors that lead to the upregulation of NKG2D ligands. Consequently, NKG2D ligands are highly expressed in multiple tumors of different origins, though in varying proportions [12]. Thus IL-2 can potentially be linked to NKG2D for its specific delivery to the tumor loci.

For the current study, we have generated a chimeric molecule linking murine IL-2 to the extracellular domain of murine NKG2D with an IgG2a Fc region (to facilitate purification) to form the chimeric NKG2D-Fc-IL2 protein. Here, we show that NKG2D-Fc-IL2 was able to bind to murine NKG2D ligand-expressing tumor cells. Furthermore, the IL-2 component of the chimeric protein was capable of inducing proliferation of T cells *in vitro* and at the tumor site *in vivo*. More importantly, TC-1 tumor-bearing mice treated with a therapeutic HPV type 16 E7 DNA vaccine and then given the DNA construct encoding the chimeric NKG2D-Fc-IL2 protein demonstrated reduced tumor mass growth and prolonged survival. Thus the data suggest the chimeric NKG2D-Fc-IL2 technology has potential to be used to further enhance the therapeutic anti-tumor effect generated by the therapeutic HPV DNA vaccine. Potential translational and clinical applications of this approach will be discussed.

## Materials and Methods

### Ethics Statement

This study was carried out in strict accordance with the recommendations of the Guide for the Care and Use of Laboratory Animals of the National Institutes of Health. All procedures were performed with prior approval of the Johns Hopkins Animal Care and Use Committee (protocol MO08M446).

### Mice

Six- to eight-week-old female C57BL/6 mice were purchased from the National Cancer Institute (Frederick, MD) and housed in the Johns Hopkins Oncology Center Animal Facility located in Cancer Research Building II (Johns Hopkins School of Medicine, Baltimore, MD). All animal procedures were performed according to approved protocols and in accordance with recommendations for the proper use and care of laboratory animals.

### Cells

TC-1 cells were produced in our laboratory and maintained as previously described [13]. Ovarian surface epithelial carcinoma (MOSEC) [14] cells were provided by Dr. Katherine Roby (University of Kansas Medical Center). The generation of the luciferase-expressing E7 (aa49-57)-specific T cell line has also been previously described [15]. Baby hamster kidney (BHK)-21 cells were obtained from ATCC (Rockville, MD). Cell lines were cultured *in vitro* in RPMI 1640 supplemented with 10% fetal bovine serum, 50 units/ml of penicillin/streptomycin, 2 mM L-glutamine, 1 mM sodium pyruvate, and 2 mM non-essential amino acids, and grown at 37°C with 5% CO_2_.

### Plasmid DNA Constructs and Preparation

pFuse-Fc (pFuse-mIgG2a-Fc2) was obtained from Invivogen (San Diego, USA). To generate pFuse-NKG2D-Fc, the extracellular domain of murine NKG2D was PCR amplified by primers (aaaGAATTCGaaagagacgtttcagccagt and tttAGATCTcaccgcccttttcatgcaga) with mouse NKG2D cDNA as the template DNA (Open Biosystems, Lafayette CO), and then cloned into EcoRI and Bgl II sites of pFuse-IgG2a (Invivogen). To clone pFuse-NKG2D-Fc-GLuc, the GLuc gene was amplified by PCR using primers (AAATCTAGAgaggccaagcccaccgagaac and aaaCTCGAGttagtcaccaccggccccctt) and cloned into the XbaI/XhoI sites of pFuse-NKG2D. The same process was employed to construct pFuse-Fc-GLuc using pFuse-Fc instead of pFuse-NKG2D. For pFuse-NKG2D-FC-IL2, IL-2 was PCR amplified using primers (aaatctagaGCACCCACTTCAAGCTCCACT and aaaCTCGAGttattgagggcttgttgaga) with a murine pcDNA3-IL2 construct as a template [16], and then cloned into XbaI/XhoI sites of pFuse-NKG2D-Fc. pFuse-Fc-IL2 was constructed using the PCR product of IL-2 cloned into the XbaI/XhoI sites of pFuse-Fc. Schematic diagram of the various chimeric genes encoded by the DNA constructs is depicted in **Figure S1**.

### Transfection and Protein Purification

For the production of the recombinant protein NKG2D-Fc-IL2 and control proteins IgG2a Fc (hereinafter “Con-Fc”), Con-Fc-GLuc, NKG2D-Fc, NKG2D-Fc-GLuc, Con-Fc-IL2, 1×l0^7^ BHK-21 cells were transfected with 50μg of each plasmid in T-150 flasks using Lipofectamin 2000 (Invitrogen Corp., Carlsbad, CA, USA). After 3 days, the cell-cultured media was accumulated, filtered with a 0.22μm syringe filter (Millipore, Billerica MA, USA) and concentrated with Amicon Ultra-15 50kDa cut-off centrifugal filter units (Millipore, Billerica MA, USA). The concentrated recombinant proteins were loaded onto a HiTrap Protein G HP column (GE Healthcare) and immobilized via Fc-protein G binding. The column was washed with 20mM sodium phosphate buffer (pH 7.0) and the recombinant protein was eluted using 0.1M glycine-Cl buffer (pH 2.8). Protein concentrations were determined with the Coomassie Plus protein assay (Pierce, Rockford, USA) and purity was estimated by SDS polyacrylamide gel electrophoresis.

### DNA Vaccination and Electroporation-mediated Intramuscular Injection

DNA-coated gold particle-mediated DNA vaccination was performed using a helium-driven gene gun (BioRad Laboratories, Inc., Hercules, CA, USA) as described in [17]. CRT/E7-encoding DNA-coated gold particles were delivered to the shaved abdominal region of mice using a helium-driven gene gun (BioRad Laboratories, Inc.) with a discharge pressure of 400 psi. C57BL/6 mice were immunized with 2μg of the CRT/E7 plasmid three times at 3-day intervals.

The pCon-Fc, pCon-Fc-GLuc, pNKG2D-Fc, pNKG2D-Fc-GLuc, pCon-Fc-IL2, and pNKG2D-Fc-IL2 plasmids were delivered intramuscularly by syringe needle injection. The specifics of the procedure are similar to those found in [18], [19]. In short, hair around the quadriceps femoris muscle of mice was removed and 40μg of plasmid DNA was injected. Immediately following the injection two-needle array electrodes (BTX, San Diego, CA) were inserted with a separation distance of 5 mm with the array inserted longitudinally relative to the fibers. Electric pulses were generated using a square-wave electroporator (Model 830; BTX). *In vivo* electroporation parameters were as follows: distance between the electrodes, 100 V/cm; pulse duration, 50 ms; 10 pulses with reversal of polarity.

### Flow Cytometry Analysis

For *in vitro* flow cytometry analysis, samples of 2x10^5^ tumor cells were incubated with 0.5μg purified recombinant protein or anti-mouse Rae-1 antibody (BD Bioscience). PE-conjugated anti-mouse Fc secondary antibody (BD Bioscience) was used as a detection antibody. To confirm that the chimeric protein could be generated and released *in vivo*, pCon-FC, pNKG2D-Fc, pCon-Fc-IL2, or pNKG2D-Fc-IL2 plasmids were intramuscularly injected followed by electroporation into naïve mice (3 mice/group). After 2 days, blood sera from mice were collected and diluted 1:50 for TC-1 cell staining. Again, PE-conjugated anti-mouse Fc secondary antibody was used as a detection antibody (BD Bioscience). CELLQuest software (Becton Dickinson Immunocytometry System, Mountain View, USA) was used for FACScan analysis with methods similar to those previously described [17].

### Luciferase-based Bioluminescence Imaging

*Gaussia* luciferase (GLuc) [20] and the substrate coelenterazine (Sigma) were used to test for GLuc activity *in vitro* and *in vivo*. For *in vitro* cell staining, TC-1 cells were added to 24-well plates at 1×10^5^ cells/well and incubated overnight. The next day, 1μg of recombinant Con-Fc, Con-Fc-GLuc, NKG2D-Fc, or NKG2D-Fc-GLuc protein was added into each well and incubated for 20 minutes at 37°C. The wells were washed twice with PBS before coelenterazine was added. Bioluminescence of the cells was detected via the IVIS Imaging System 200 Series. The region of interest from displayed images was designated and quantified as total photon counts using Living Image 2.50 software (Xenogen). For the *in vivo* luciferase attraction experiment, mice were injected with 1×10^5^ TC-1 cells. After 10 days, pCon-Fc-GLuc or pNKG2D-Fc-GLuc plasmid was intramuscularly injected followed by electroporation. The following day, coelenterazine was intraperitoneally injected and the bioluminescence of the cells was detected as described above. For the *in vitro* cell staining and T-cell proliferation experiments, 5×10^4^ irradiated MOSEC cells were added to 48-well plates and incubated overnight. The following day, recombinant Con-Fc, NKG2D-Fc, Con-Fc-IL2, or NKG2D-Fc-IL2 protein was added and incubated for 30 min in 37°C. After washing twice with PBS, 1×10^5^ luciferase-expressing E7-specific CD8+ T cells was added to each well and allowed to incubate for five days. The levels of light from the bioluminescent cells were detected as described above. For the *in vivo* T cell attraction experiment, mice were injected with 1×10^5^ TC-1 cells. After ten days, 40μg pCon-Fc, pNKG2D-Fc, pCon-Fc-IL2, or pNKG2D-Fc-IL2 plasmid was injected using intramuscular injection followed by electroporation and 5x10^6^ luciferase-expressing E7-specific CD8+ T cells were adoptively transferred intravenously through the tail vein into the mice. Five days after injection, the bioluminescence of the cells was detected as described above.

### *In Vivo* Tumor Treatment Experiments

For the TC-1 tumor treatment experiment, naïve mice were subcutaneously challenged with 1×l0^5^ TC-1 tumor cells/mouse. After six days, the mice were vaccinated using the procedure described above with pCRT/E7 via gene gun, followed by plasmids of pCon-Fc, pNKG2D-Fc, pCon-Fc-IL2, or pNKG2D-Fc-IL2 using electroporation-mediated intramuscular injection three times at 3-day intervals starting 12 days after vaccination. Tumor growth was monitored by palpation and inspection twice a week until the mice died.

### Statistical Analysis

Experiments were performed in duplicate or triplicate. The data presented here are of one representative experiment, and are expressed as means ± standard deviation (S.D.). The number of samples in each group for any given experiment was >5. Results for intracellular cytokine staining with flow cytometry analysis and tumor treatment experiments were evaluated by analysis of variance (one-way ANOVA) and the Turkey-Kramer multiple comparison test. Comparisons between individual data points were performed using Student’s t-test. The event time distributions for different mice were compared using the Kaplan-Meier method and the log-rank statistic. All p values < 0.05 were considered significant.

## Results

### The NKG2D-Fc Protein Binds to Tumor Cells Expressing Murine NKG2D Ligands

**Figure 1:**
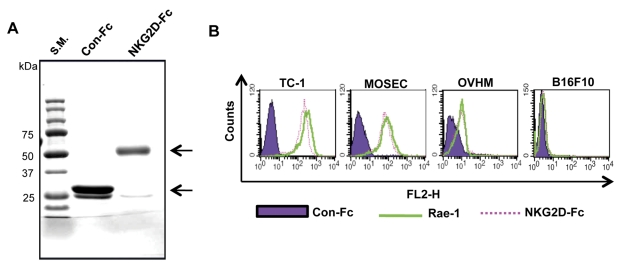
Characterization of the murine NKG2D-Fc protein. (A) Gel electrophoresis was used to characterize the size of the purified NKG2D-Fc and Con-Fc proteins. The NKG2D-Fc and Con-Fc proteins were purified from BHK-21 cells transfected with the pFuse-Fc and pFuse-NKG2D-Fc DNA constructs. The purity and size of isolated protein was characterized by SDS-PAGE, followed by staining with Coomassie brilliant blue. The arrows indicate the proteins of interest. (B) Characterization of the binding of NKG2D-Fc to Rae-1 expressed in different tumor cells using flow cytometry.

We have successfully isolated the NKG2D-Fc and Con-Fc proteins from the DNA-transfected BHK-21 cells and used SDS-PAGE to assess their size and purity, as shown in **Figure 1A**. The stained gel shows bands of high purity and of the expected size for both proteins.

To determine if the purified NKG2D-Fc could bind to murine NKG2D ligand-expressing tumor cells, we selected three NKG2D ligand-expressing tumor cell lines (TC-1, MOSEC, and OVHM; all express Rae-1, a murine NKG2D ligand) and one Rae-1-negative tumor cell line (B16F10). As shown in **Figure 1B**, NKG2D-Fc bound to all three of the NKG2D ligand-expressing tumor cell lines, but not to the Rae-1-negative tumor cells. This is consistent with previous reports that cells engineered to express the extracellular domain of murine NKG2D are capable of recognizing and binding to cells NKG2D ligand-expressing cells, including Rae-1-expressing cells [21]. We further demonstrated that NKG2D-Fc bound to Rae-1-expressing tumor cells through the NKG2D component, rather than the Fc component, since Con-Fc (which does not have the NKG2D component) failed to generate significant binding (see **Figure S2**). Taken together, these data suggest NKG2D-Fc is capable of binding NKG2D ligand-expressing tumor cells.

### The Linkage of NKG2D-Fc to *Gaussia* Luciferase (GLuc) Lends Confirmation that the Constructed Chimeric Molecule Targets the Tumor Loci in Tumor-bearing Mice

**Figure 2:**
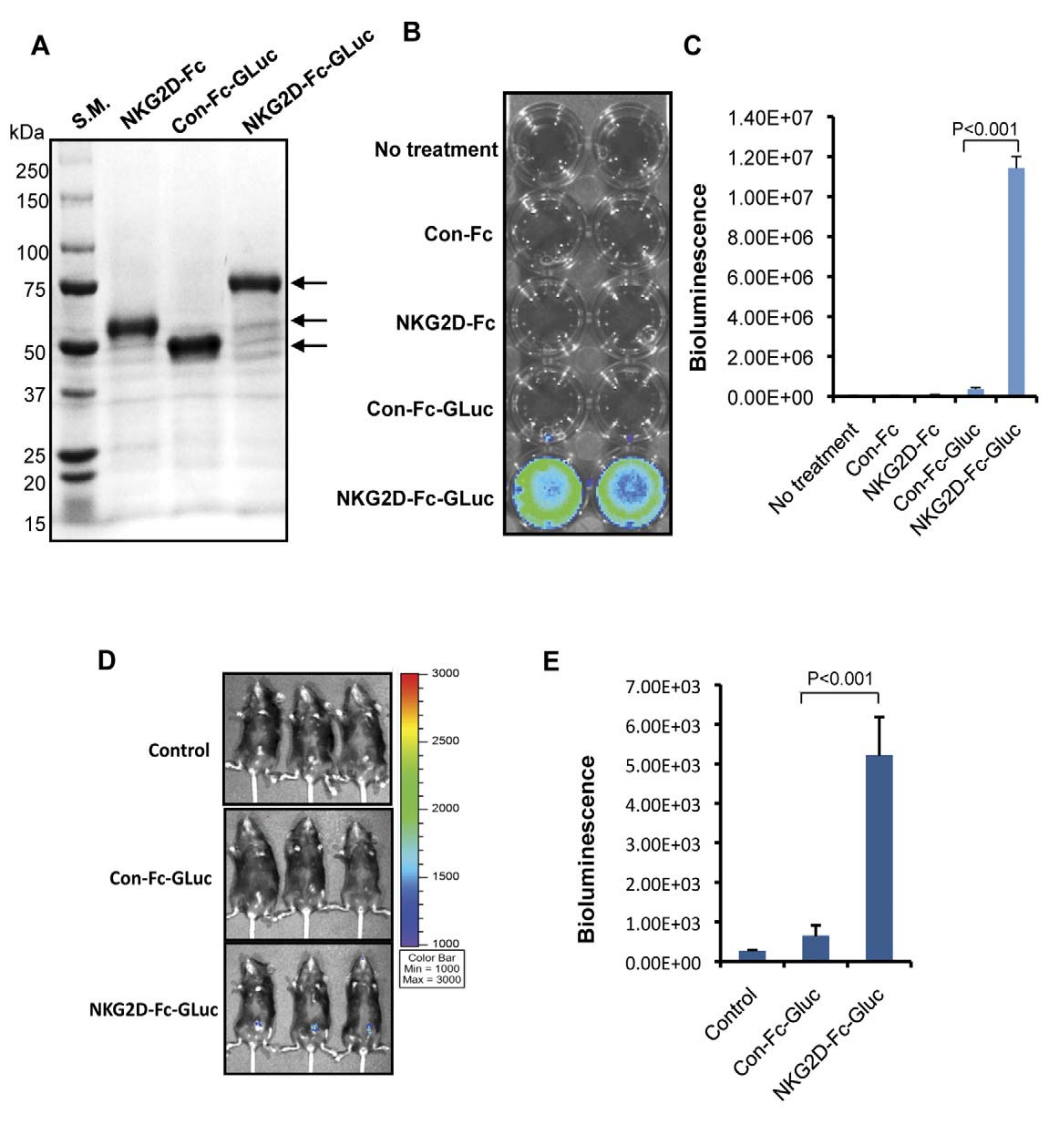
Characterization and luminescence activity of NKG2D-Fc-GLuc protein. (A) Gel electrophoresis was used to characterize the size of the purified NKG2D-Fc-GLuc, Con-Fc-GLuc, and NKG2D-Fc proteins. The purity and size of the isolated proteins were characterized by SDS-PAGE, followed by staining with Coomassie brilliant blue. The arrows indicate the proteins of interest. (B) Representative figure of bioluminescent imaging in TC-1 cells incubated with the different purified proteins. TC-1 cells were plated into a 24-well plate. The purified proteins (Con-Fc, NKG2D-Fc, Con-Fc-GLuc, and NKG2D-Fc-GLuc) were added to the TC-1 tumor cells. The binding of NKG2D-Fc-GLuc to TC-1 cells was characterized by bioluminesence imaging using the IVIS Imaging System 200 Series. (C) Histogram depicting the bioluminescence measured in Figure 2B. The p-value found was less than 0.001. (D) Representative bioluminescence imaging to demonstrate the specific targeting of NKG2D-Fc-GLuc to TC-1 tumor cells *in vivo*. TC-1 tumor-bearing C57BL/6 mice were injected intramuscularly with the DNA constructs encoding the Con-Fc-GLuc or NKG2D-Fc-GLuc protein, followed by electroporation. The treated mice were imaged for bioluminescence using the IVIS Imaging System 200 Series. (E) Histogram of the bioluminescence imaging found in Figure 2D. Note: there is a significant difference in the bioluminescence of the tumors between the mice injected with the DNA constructs for Con-Fc-Glu and NKG2D-Fc-Glu (P-value less than 0.001).

In order to determine if NKG2D-Fc could serve as a reliable strategy for directing the constructed protein to tumor cells, particularly to the tumor loci in tumor-bearing mice, we used *Gaussia* luciferase (GLuc) as a marker protein. We successfully generated and isolated the chimeric NKG2D-Fc-GLuc protein as well as the control protein, Con-Fc-GLuc. As shown in **Figure 2A**, we used SDS-PAGE to assess the size and purity of the proteins. The stained gel shows prominent bands of high purity and of the expected size for NKG2D-Fc-GLuc, Con-Fc-GLuc, and NKG2D-Fc.

To determine both the functionality of the *Gaussia* luciferase and the binding specificity of the NKG2D-Fc-GLuc protein to Rae-1-expressing tumor cells, we incubated NKG2D-Fc-Gluc with Rae-1-expressing TC-1 tumor cells. Equivalent assays were also completed with relevant control proteins. The treated tumor cells were washed and incubated with the substrate coelenterazine before being imaged with the IVIS Imaging System 200 Series. As shown in **Figures 2B and 2C**, only the TC-1 tumor cells treated with NKG2D-Fc-GLuc demonstrated significant luciferase activity, when compared to TC-1 tumor cells incubated with the Con-Fc-GLuc, NKG2D-Fc, or Con-Fc, or with no treatment. These data suggest that the linkage of NKG2D-Fc to *Gaussia* luciferase does not interfere with the specific binding of the chimeric protein to Rae-1-expressing tumor cells, nor does the linkage affect the enzymatic function of the *Gaussia* luciferase.

We then determined if tumor-bearing mice injected with the DNA construct encoding the NKG2D-Fc-GLuc protein would be able to target and concentrate the chimeric protein at the tumor loci of the injected mice. TC-1 tumor-bearing mice were intramuscularly injected with DNA encoding either the NKG2D-Fc-GLuc or Con-Fc-GLuc protein, followed by electroporation. Administration of coelenterazine followed, and the mice were imaged using the IVIS Imaging System 200 Series. As shown in **Figures 2D and 2E**, tumor-bearing mice injected with the NKG2D-Fc-GLuc DNA construct showed significant luminescent activity concentrated at the tumor loci. In contrast, the tumor-bearing mice injected with Con-Fc-GLuc DNA construct did not demonstrate any luciferase activity. Collectively, our data show that NKG2D-Fc linked to *Gaussia* luciferase can target and concentrate the *Gaussia* luciferase at the tumor loci in tumor-bearing mice. Thus, we have created a system to target the constructed protein to the NKG2D ligand-expressing tumor *in vivo*.

### NKG2D-Fc-IL2 is Capable of Binding to NKG2D Ligand-expressing Tumor Cells while Preserving IL-2 Functionality of Stimulating T Cell Proliferation

**Figure 3:**
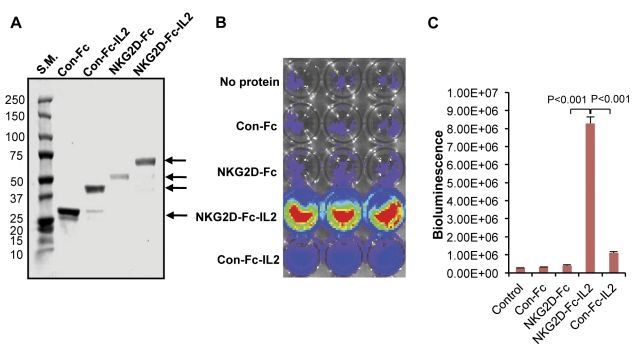
Characterization of NKG2D-Fc-IL2 protein. (A) Gel electrophoresis was used to characterize the size of the purified NKG2D-Fc-IL2, NKG2D-Fc, Con-Fc-IL2, and Con-Fc proteins. The purity and size of the isolated proteins were characterized by SDS-PAGE, followed by staining with Coomassie brilliant blue. The arrows indicate the proteins of interest. (B) Representative figure of the T-cell proliferation assay using bioluminescent imaging in irradiated MOSEC cells incubated with the different purified proteins. MOSEC cells were plated into a 24-well plate. The purified proteins (Con-Fc, NKG2D-Fc, Con-Fc-IL2, and NKG2D-Fc-IL2) were added to the MOSEC tumor cells, allowed to incubate, and then washed. Luciferase-expressing E7-specific T cells were added and incubated for five days. The proliferation of the T cells was characterized by bioluminescence imaging using the IVIS Imaging System 200 Series. (C) Histogram of the luminescence imaging cubated NKG2D-Fc-IL2 than in the wells incubated with NKG2D-Fc or Con-Fc-IL2 (P-value less than 0.001).

IL-2 has been shown to promote T cell proliferation. We reason that the specific delivery of IL-2 to the tumor loci may enhance the proliferation of tumor specific CD8+ T cells in the tumor microenvironment. In order to determine if the NKG2D-Fc protein could be used to deliver IL-2 to the tumor loci, we linked NKG2D-Fc to IL-2, generating the chimeric NKG2D-Fc-IL2 protein. We successfully generated and isolated the NKG2D-Fc-IL2 protein from DNA-transfected BHK-21 cells and used SDS-PAGE to assess the size and purity of the NKG2D-Fc-IL2 protein, as well as the relevant control proteins (NKG2D-Fc, Con-Fc-IL2, and Con-Fc). As shown in **Figure 3A**, the stained gel shows bands of high purity and of the expected size for all proteins.

In order to determine if NKG2D-Fc-IL2 can bind to NKG2D ligand-expressing tumor cells while preserving the IL-2 functionality of promoting T cell proliferation, we incubated NKG2D-Fc-IL2 with irradiated MOSEC tumor cells, alongside equivalent assays with relevant control proteins. The treated tumor cells were washed and incubated with luciferase-expressing E7-specific T cells for five days. The proliferation of T cells was measured by bioluminescence imaging using the IVIS Imaging System 200 Series. As shown in **Figures 3B and 3C**, the MOSEC tumor cells treated with NKG2D-Fc-IL2 demonstrated significantly more luciferase activity in comparison to the MOSEC tumor cells treated with Con-Fc-IL2, NKG2D-Fc, or Con-Fc, or to the control (no treatment). These data suggest that the chimeric NKG2D-Fc-IL2 protein is capable of binding to Rae-1-expressing tumor cells and the IL-2 component is functionally capable of promoting the proliferation of antigen-specific CD8+ T cells.

### TC-1 Tumor-bearing Mice Injected with DNA Encoding NKG2D-Fc-IL2 Exhibit an Increased Number of Luciferase-expressing E7-specific CD8+ T Cells at the Tumor Site

**Figure 4:**
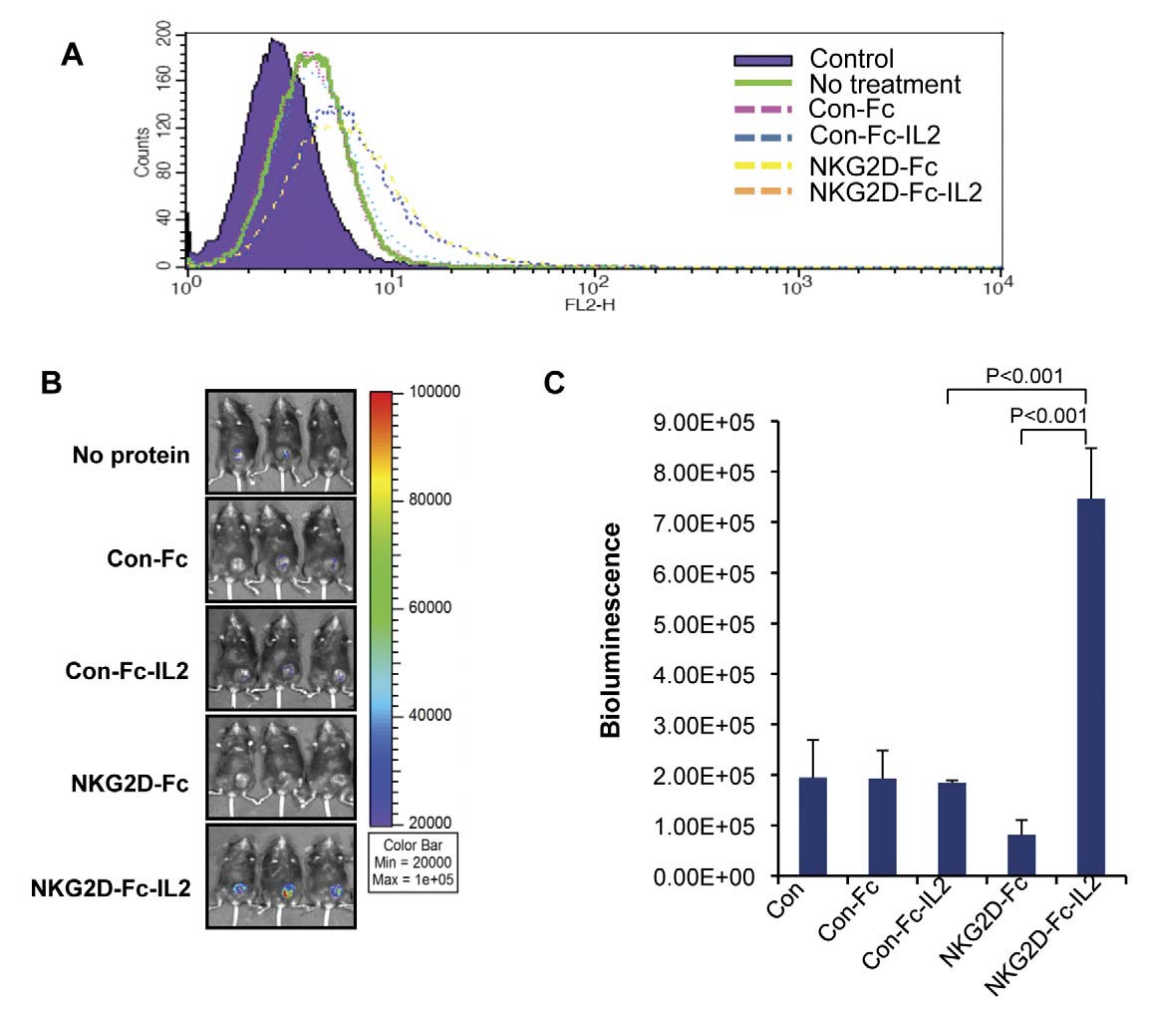
Characterization of T cell accumulation in tumor-bearing mice intramuscularly injected with DNA constructs with electroporation. (A) Characterization of the binding of NKG2D-Fc-containing protein to TC-1 tumor cell using serum from mice injected intramuscularly with the various DNA constructs in conjunction with electroporation. Mice were intramuscularly injected with one of four DNA constructs. Serum was collected from the mice and incubated with TC-1 tumor cells. The cells were incubated with anti-Fc-PE and characterized by flow cytometry. Note: the tumor cells incubated with the serum from mice injected with the DNA constructs encoding NKG2D-Fc or NKG2D-Fc-IL2 led to a significant shift compared to other constructs. (B) Representative bioluminescence imaging used to characterize the presence of luciferase-expressing E7-specific CD8+ T cells in tumor-bearing mice injected intramuscularly with different DNA constructs followed by electroporation. TC-1 tumor bearing mice were injected intramuscularly with one of the four DNA constructs, followed by electroporation. Luciferase-expressing T cells were injected intravenously into the lateral tail vein and the bioluminescence was imaged five days later. Note: the luciferase-expressing T cells proliferated locally to the tumor site in the mice injected with DNA encoding NKG2D-Fc-IL2 but not in others. (C) Histogram of the bioluminescence imaging depicted in Figure 4B. Note: the luminescence of the tumors between the mice injected with NKG2D-Fc-IL2 and those not (P-value less than 0.001).

In order to determine if (1) mice could be injected intramuscularly with the DNA construct encoding NKG2D-Fc-IL2 and successfully produce detectable amounts of the chimeric protein and (2) the protein was capable of targeting NKG2D ligand-expressing tumor cells *in vivo*, TC-1 tumor-bearing mice were intramuscularly injected with DNA constructs encoding NKG2D-Fc-IL2, NKG2D-Fc, Con-Fc-IL2, Con-Fc, or no protein, followed by electroporation. To test the production of chimeric protein, serum samples were collected from each mouse following treatment, incubated with TC-1 tumor cells *in vitro*, and prepared for flow cytometry analysis. As shown in **Figure 4A**, the samples containing NKG2D-Fc and NKG2D-Fc-IL2 produced shifts significantly different from the TC-1 tumor cells treated with Con-Fc or Con-Fc-IL2, demonstrating that intramuscular injection of a DNA construct encoding the proteins consisting of NKG2D-Fc followed by electroporation can produce detectable amounts of functional chimeric protein in the serum.

We further determined that the TC-1 tumor-bearing mice treated with the DNA construct encoding NKG2D-Fc-IL2 could exhibit enhanced proliferation of the luciferase-expressing E7-specific CD8+ T cells at the tumor loci. After injection with the DNA construct followed by electroporation, the TC-1 tumor-bearing mice were injected with luciferase-expressing E7-specific CD8+ T cells. After five days, bioluminescence imaging of the mice was performed using the IVIS Imaging System 200 Series. As shown in **Figures 4B**, the tumor-bearing mice injected with NKG2D-Fc-IL2 showed significant luciferase activity at the tumor loci. In contrast, the other treated mice showed no significant luciferase activity. Taken together, our data show that TC-1 tumor-bearing mice injected with the DNA construct encoding NKG2D-Fc-IL2 are capable of delivering IL-2 to the tumor loci, resulting in local proliferation and accumulation of E7-specific CD8+ T cells.

### Treatment with DNA Encoding NKG2D-Fc-IL2 Significantly Enhanced the Therapeutic Anti-tumor Effects Generated by Intradermal Vaccination with Therapeutic HPV DNA in Tumor-bearing Mice

**Figure 5:**
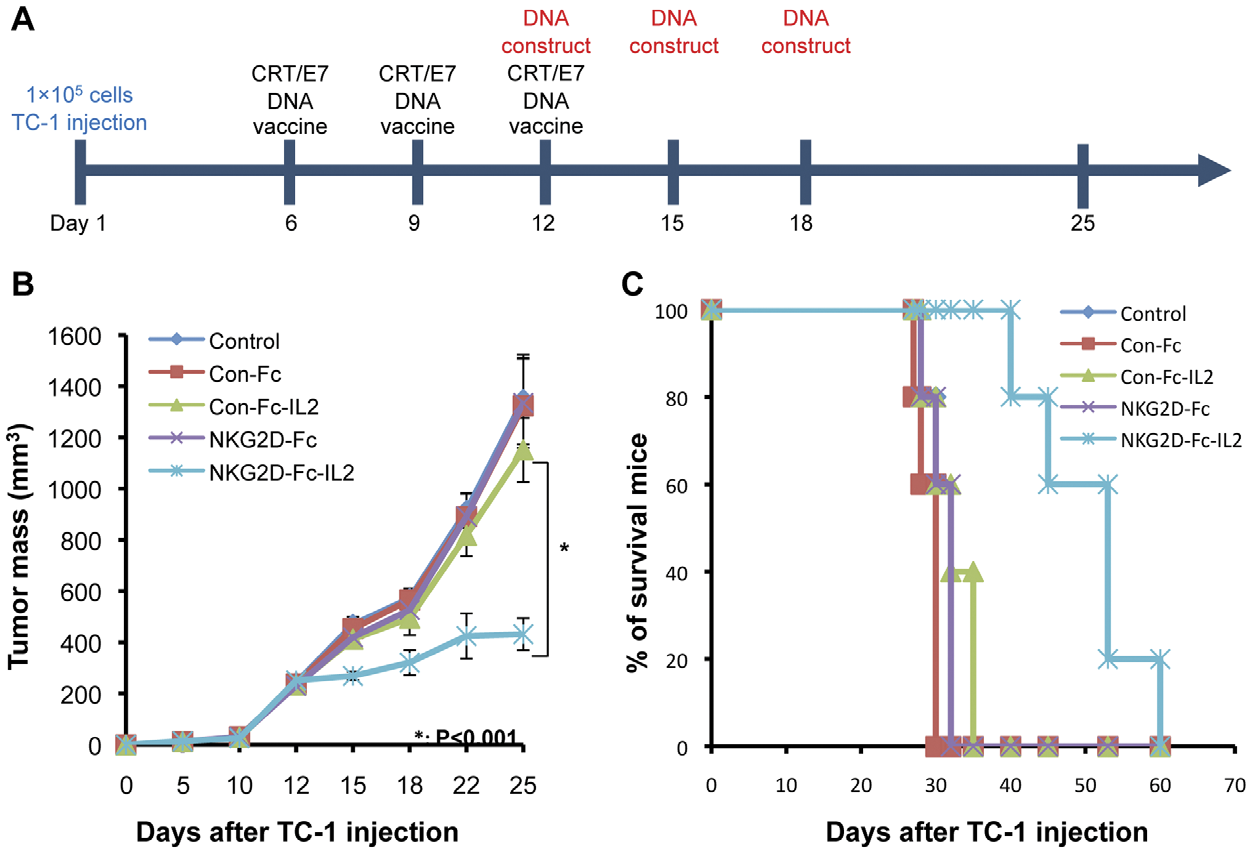
Therapeutic anti-tumor effects of intramuscular injection of different DNA constructs in vaccinated tumor-bearing mice. (A) Schematic diagram depicting the regimen. On Day 1, TC-1 cells were injected subcutaneously. Five days later, the mice were vaccinated with the CRT/E7 DNA vaccine via gene gun, with boosters of the same dose given on Days 9 and 12. On Days 12, 15, and 18, the mice were intramuscularly injected with one of the DNA constructs, followed by electroporation. (B) Line graph illustrating the changes in tumor size in the TC-1 tumor-bearing mice over time. The dimensions of the tumor were characterized by measurement. (C) Kaplan-Meier survival analysis of the tumor-bearing mice following the TC-1 tumor challenge and treatment.

We have previously demonstrated that the intradermal administration of a DNA vaccine encoding Calreticulin linked to E7 (CRT/E7) via gene gun was able to generate high numbers of E7-specific CD8+ T-cell immune responses and therapeutic anti-tumor effects in vaccinated mice [22]. Therefore, in the current study we determined if the anti-tumor effects in tumor-bearing mice receiving the CRT-E7 DNA vaccine could be enhanced by additional treatment with the DNA construct encoding NKG2D-Fc-IL2. The tumor-bearing mice were first treated with the CRT/E7 DNA vaccine via gene gun and later received an intramuscular injection of the DNA construct encoding NKG2D-Fc-IL2 followed by electroporation (see **Figure 5A** for a schematic diagram of the procedure). As shown in **Figure 5B**, the vaccinated tumor-bearing mice receiving the NKG2D-Fc-IL2 construct demonstrated a significant reduction in tumor mass growth, when compared to the other groups of mice receiving NKG2D-Fc, Con-Fc-IL2, or Con-Fc, or the DNA vaccine alone. Furthermore, as shown in **Figure 5C**, the tumor-bearing mice receiving the NKG2D-Fc-IL2 construct exhibited prolonged survival, in comparison the other tumor-bearing mice treated with the other DNA constructs. Thus, our data suggest the additional administration of the DNA construct encoding NKG2D-Fc-IL2 is able to enhance the therapeutic anti-tumor effect first generated by the gene gun-administered CRT-E7 DNA vaccine.

## Discussion

In this study, we demonstrated that a constructed protein, NKG2D-Fc, was able to bind to murine NKG2D ligand-expressing tumor cells. Furthermore, we were able to use the NKG2D-Fc protein to deliver both a marker protein (GLuc) and IL-2 to the tumor loci in tumor-bearing mice. More importantly, we demonstrated that IL-2 linked to NKG2D-Fc led to significant proliferation of the tumor-specific CD8+ T-cell at the tumor loci and resulted in potent therapeutic anti-tumor effects in TC-1 tumor-bearing mice that received the therapeutic HPV16/E7 DNA vaccine. Thus, our study demonstrated that constructed proteins containing NKG2D are capable of specifically targeting NKG2D ligands, often found to be overexpressed on tumor cells, making the NKG2D-ligand system an exploitable receptor-ligand system to bring potentially immune-modulating or therapeutic molecules to the tumor site.

The success of this approach, using NKG2D ligands as specific targets highly expressed in many tumor cells, warrants further exploration of other molecules that are also uniquely expressed on the surface of tumor cells, but not found on normal tissue. One example is mesothelin – a protein also commonly overexpressed on many human cancer cells, including ovarian [23], [24] and pancreatic cancer [24]. Other potential target molecules for targeting ovarian cancer cells might include follicle-stimulating hormone receptor (FSHR) [25], intercellular adhesion molecule 1 (ICAM-1) [26], Müllerian inhibiting substance type II receptor (MISIIR) [27], or human epidermal growth factor receptor 2 (HER2) [28]. Prostate-specific membrane antigen (PSMA) is expressed in orders of magnitude greater on prostate tumor cells than normal cells [29], therefore making PSMA a potential target molecule for treating prostate cancer. With contemporary technologies including deep sequencing, gene and protein microarrays, proteomics, and many others, we expect to see more potential candidate targets emerge in the near future. If these candidate targets are, in actuality, uniquely expressed in cancer cells but not in normal tissues, these targets can potentially be used to deliver specific molecules to the tumor loci using its counterpart through a receptor-ligand system or an antibody-antigen system.

The NKG2D-Fc system also allows us to deliver other immune-modulating or anti-cancer molecules (i.e., other than IL-2) to the tumor loci with the added benefit of reduced toxicity because of its specific targeting capabilities. One potential concern with the delivery of IL-2 to the tumor loci is the expansion of regulatory T (Treg) cells. However, with the current study, we have shown that the specific delivery of IL-2 with the NKG2D-Fc system led to both the expansion of tumor antigen-specific CD8+ T cells at the tumor loci and an improved therapeutic anti-tumor effect generated by the therapeutic DNA vaccine. Thus, our data suggest the expansion of Treg cells by the local delivery of IL-2 does not significantly impede the therapeutic anti-tumor effect generated by the therapeutic HPV DNA vaccine. Some other potential candidates for anti-cancer molecules are molecules that can activate immune responses in the tumor microenvironment, such as GM-CSF (granulocyte macrophage colony-stimulating factor) or IL-12. IL-15 has even been shown to be more effective than IL-2 for tumor growth inhibition [5]. The use of these molecules could relieve the concerns of Treg expansion. Molecules capable of countering the immunosuppressive factors in the tumor microenvironment are also worth further investigation, as are molecules capable of directly leading to tumor cell death or growth inhibition, such as *Pseudomonas* exotoxin-A or other bacterial toxins.

One potential limitation for the clinical translation of the proposed approach is antibody generation, specifically against the chimeric protein. If antibodies are generated to the chimeric protein, subsequent challenges may be affected. Therefore, as a step towards clinical translation, it will be important to use the human counterparts for the murine IL-2, Fc, and NKG2D of this study to generate a clinical grade reagent such that the immune response against the chimeric protein will be limited. It will also be important to test the binding ability of the human NKG2D-Fc-IL2 to different human cancer cell lines, as well as characterize the functionality of the IL-2 component before considering the generation of a clinical grade reagent for clinical trials.

In summary we have successfully developed a strategy to specifically target anti-cancer molecules to tumor loci. Such a strategy can potentially be used to deliver various anti-cancer molecules to the tumor loci for therapeutic anti-tumor effects. Since NKG2D ligands are highly expressed in many different tumor cells, our strategy represents a potentially useful approach for delivering molecules of interest to the tumor loci for the control of different kinds of tumors for future clinical translation.

## Supporting Information

Figure S1 **Structure of the various chimeric genes.** Schematic diagram to illustrate the composition of the various chimeric genes used in DNA constructs
(TIF)

Figure S2 **NKG2D-Fc binds to Rae-1 expressing tumor cells through the NKG2D component.** Flow cytometry to characterize the binding of NKG2D-Fc and Con-Fc to tumor cells. TC-1 and MOSEC cell lines were incubated with either purified Con-Fc or NKG2D-Fc proteins followed by a stain using secondary phycoerythrin-labeled (PE) antibody against Fc (anti-Fc-PE). Note that the larger shifts for NKG2D-Fc show that NKG2D-Fc can bind to both the TC-1 and MOSEC cell lines. On the other hand, Con-Fc generates only a minor shift in comparison to the control.
(TIF)
