# Supplementary figures and images for: Tumor-Targeted Delivery of IL-2 by NKG2D Leads to Accumulation of Antigen-Specific CD8+ T Cells in the Tumor Loci and Enhanced Anti-Tumor Effects
Source: PLoS One. 2012 Apr 11;7(4):e35141. doi: 10.1371/journal.pone.0035141 (PMC3324421; doi:10.1371/journal.pone.0035141)

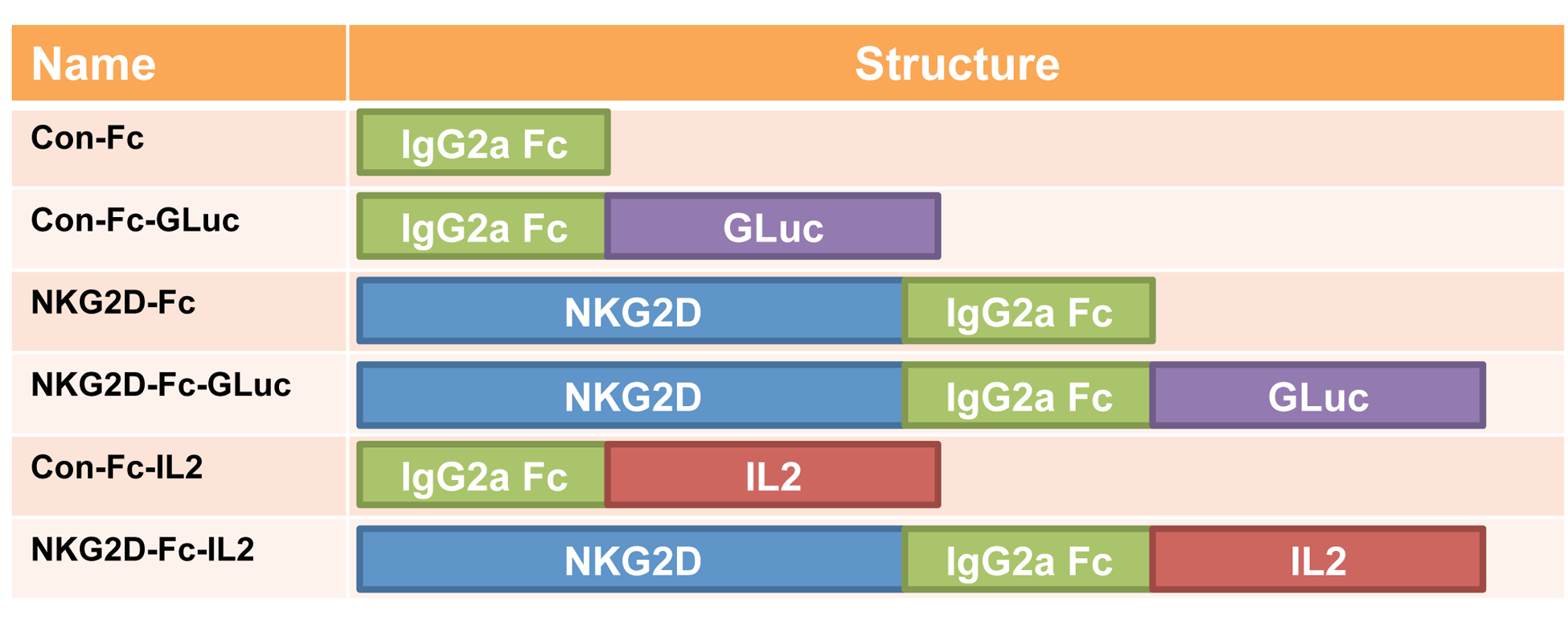

Supplement: Figure S1 — Structure of the various chimeric genes. Schematic diagram to illustrate the composition of the various chimeric genes used in DNA constructs (TIF) [file pone.0035141.s001.tif]

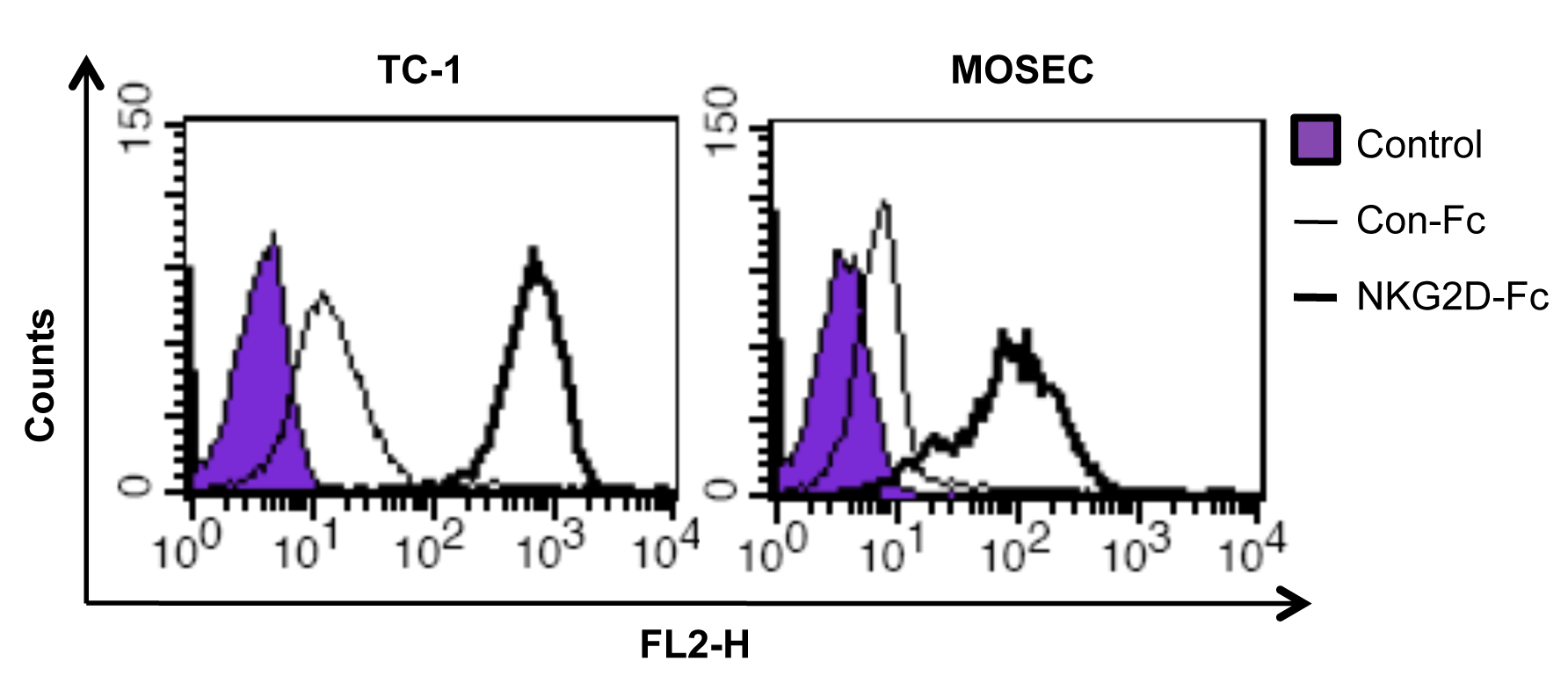

Supplement: Figure S2 — NKG2D-Fc binds to Rae-1 expressing tumor cells through the NKG2D component. Flow cytometry to characterize the binding of NKG2D-Fc and Con-Fc to tumor cells. TC-1 and MOSEC cell lines were incubated with either purified Con-Fc or NKG2D-Fc proteins followed by a stain using secondary phycoerythrin-labeled (PE) antibody against Fc (anti-Fc-PE). Note that the larger shifts for NKG2D-Fc show that NKG2D-Fc can bind to both the TC-1 and MOSEC cell lines. On the other hand, Con-Fc generates only a minor shift in comparison to the control. (TIF) [file pone.0035141.s002.tif]
